# Obesity and Its Impact on Adverse In-Hospital Outcomes in Hospitalized Patients With COVID-19

**DOI:** 10.3389/fendo.2022.876028

**Published:** 2022-05-02

**Authors:** Karsten Keller, Ingo Sagoschen, Volker H. Schmitt, Visvakanth Sivanathan, Christine Espinola-Klein, Carl J. Lavie, Thomas Münzel, Lukas Hobohm

**Affiliations:** ^1^ Department of Cardiology, University Medical Center of the Johannes Gutenberg-University Mainz, Mainz, Germany; ^2^ Center for Thrombosis and Hemostasis (CTH), University Medical Center of the Johannes Gutenberg-University Mainz, Mainz, Germany; ^3^ Medical Clinic VII, Department of Sports Medicine, University Hospital Heidelberg, Heidelberg, Germany; ^4^ German Center for Cardiovascular Research (DZHK)Partner Site Rhine Main, Mainz, Germany; ^5^ Department of Gastroenterology, University Medical Center Mainz (Johannes Gutenberg-University Mainz), Mainz, Germany; ^6^ Department of Cardiovascular Disease, John Ochsner Heart & Vascular Institute, Ochsner Clinical School, The University of Queensland School of Medicine, New Orleans, LA, United States

**Keywords:** COVID-19, human resources, obesity, ventilation, intensive and critical care

## Abstract

**Background:**

An increasing level of evidence suggests that obesity not only is a major risk factor for cardiovascular diseases (CVDs) but also has adverse outcomes during COVID-19 infection.

**Methods:**

We used the German nationwide inpatient sample to analyze all hospitalized patients with confirmed COVID-19 diagnosis in Germany from January to December 2020 and stratified them for diagnosed obesity. Obesity was defined as body mass index ≥30 kg/m^2^ according to the WHO. The impact of obesity on in-hospital case fatality and adverse in-hospital events comprising major adverse cardiovascular and cerebrovascular events (MACCE), acute respiratory distress syndrome (ARDS), venous thromboembolism (VTE), and others was analyzed.

**Results:**

We analyzed data of 176,137 hospitalizations of patients with confirmed COVID-19 infection; among them, 9,383 (5.3%) had an additional obesity diagnosis. Although COVID-19 patients without obesity were older (72.0 [interquartile range (IQR) 56.0/82.0] vs. 66.0 [54.0/76.0] years, p < 0.001), the CVD profile was less favorable in obese COVID-19 patients (Charlson comorbidity index 4.44 ± 3.01 vs. 4.08 ± 2.92, p < 0.001). Obesity was independently associated with increased in-hospital case fatality (OR 1.203 [95% CI 1.131–1.279], p < 0.001) and MACCE (OR 1.168 [95% CI 1.101–1.239], p < 0.001), ARDS (OR 2.605 [95% CI 2.449–2.772], p < 0.001), and VTE (OR 1.780 [95% CI 1.605–1.973], p < 0.001) and also associated with increased necessity of treatment on intensive care unit (OR 2.201 [95% CI 2.097–2.310], p < 0.001), mechanical ventilation (OR 2.277 [95% CI 2.140–2.422], p < 0.001), and extracorporeal membrane oxygenation (OR 3.485 [95% CI 3.023–4.017], p < 0.001).

**Conclusions:**

Obesity independently affected case fatality, MACCE, ARDS development, VTE, and other adverse in-hospital events in patients with COVID-19 infection. Obesity should be taken into account regarding COVID-19 prevention strategies, risk stratification, and adequate healthcare planning. Maintaining a healthy weight is important not only to prevent cardiometabolic diseases but also for better individual outcomes during COVID-19 infection.

## Introduction

The first severe acute respiratory syndrome coronavirus 2 (SARS-CoV-2) infection in Germany was reported on January 27, 2020 (1, 2), about 2 months after the first pneumonia patient-cases of unknown origin were identified in Wuhan, China (3, 4). Patients with detected SARS-CoV-2 infection have been documented both in hospitals and in family settings (4). The first reports from China indicated mild symptoms in the majority (approximately 4/5) of patients with SARS-CoV-2 infection, about one-fifth were hospitalized, and among them, one-fourth were admitted to intensive care units (ICUs) (3, 5, 6). From China, the SARS-CoV-2 pandemic spread worldwide and affected nearly every country (1, 5, 7, 8). In order to avoid a critical overload of the healthcare system, many countries, like Germany, have implemented lockdown strategies (5, 8, 9). Since the beginning of the COVID-19 pandemic, deaths related to COVID-19 counted more than 5 million people worldwide (10). In particular, the case-fatality rate of COVID-19 patients who have to be treated in ICUs with mechanical ventilation (MV) is very high (2, 5, 9). Studies have shown an association between COVID-19 and cardiovascular disease (CVD) (11, 12). Particularly, preexisting CVD seems to be linked with poor outcomes in patients with COVID-19 (11, 12). Although the predominant clinical manifestation of COVID-19 is pneumonia, COVID-19 infection can also induce CVD such as acute coronary syndrome with myocardial injury, arrhythmia, and venous thromboembolism (VTE) (2, 11, 12). Vascular response to cytokine production of SARS-CoV-2 and interaction between severe acute respiratory syndrome in COVID-19 and angiotensin-converting enzyme 2 receptor might lead to a significant reduction regarding cardiac contractility and result in myocardial dysfunction (12).

Another ongoing pandemic is the increase of obesity worldwide (13, 14). Obesity is a major healthcare concern, not only in high-income countries but even in middle-income and low-income countries, because of its continuous increase in the populations and of its association with chronic diseases, such as CVD, diabetes mellitus (DM), chronic kidney disease (CKD), and some cancer entities (13, 14). In the context of the COVID-19 pandemic, many studies (but not all) identified obesity as a strong risk factor for adverse outcomes in patients with SARS-CoV-2 infection (2, 13, 15–21).

Obesity is accompanied by a well-known chronic inflammatory condition (21). Obesity and its effects on immunity might aggravate disease severity of pneumonia and acute respiratory distress syndrome (ARDS), which are important causes of death due to SARS-CoV-2 infection (21). Adipocytes of the adipose tissue produce and secrete the hormone leptin in proportion to individuals’ body fat mass (21). Increasing levels of circulating plasma leptin are typical for obesity and associated with a leptin-resistant state (21). Leptin, which regulates appetite and immunity, functions in immunity as a cytokine coordinating a host’s innate as well as adaptive responses by promoting the Th1 type of the immune response (21). Leptin is an important factor regarding proliferation and different functions of antigen-presenting cells, T-helper cells, and monocytes, subsequently influencing the pro-inflammatory cytokine secretions by these cells including TNF-a, IL-2, and/or IL-6 (21). Scarcity of leptin levels and leptin resistance correlate with dysregulation of cytokine secretion resulting in autoimmune disorders, inflammatory responses, and especially increased susceptibility towards infections (21, 22). Thus, higher leptin levels and leptin activity in obese individuals contribute to higher mortality rates during SARS-CoV-2 infection (21, 22). In addition, the increased susceptibility to SARS-CoV-2 infection documented in obesity suggests an initial defect in the defense mechanisms, most likely caused by the aforementioned higher systemic metabolic inflammation, which is regulated by NLRP3 inflammasome as a master regulator of metaflammation with a pivotal role in obesity (23–25). NLRP3 inflammasome over-activation contributes to the development of cardiometabolic disorders, while NLRP3 deficiency is accompanied by decreased immune cell activation and, therefore, plays a key role in the immune defense of the host against pathogens, including viruses (23–25). Moreover, increased abdominal visceral adiposity compromises pulmonary function, decreases diaphragmatic excursion, and impairs lung ventilation resulting in reduced oxygen saturated blood levels (22).

The objectives of the present study were to investigate differences in patient characteristics, treatments, and adverse in-hospital events and outcomes of COVID-19 patients with and without obesity as well as the impact of obesity on adverse in-hospital events and outcomes of COVID-19 patients in Germany.

## Methods

### Data Source

The statistical analyses of this study were performed on our behalf by the Research Data Center (RDC) of the Federal Bureau of Statistics (Wiesbaden, Germany). Aggregated statistical results were provided by the RDC on the basis of generated SPSS codes (IBM Corp. Released 2011. IBM SPSS Statistics for Windows, Version 20.0. IBM Corp: Armonk, NY, USA), which we had created and sent to the RDC (source: RDC of the Federal Statistical Office and the Statistical Offices of the federal states, DRG Statistics 2020, own calculations) (2, 26, 27).

In the present data study-analysis of the German nationwide inpatient sample, we aimed to investigate temporal trends of all hospitalized patients with a confirmed COVID-19 diagnosis (ICD code U07.1) during the observational period between January 1 and December 31, 2020, and stratify the included COVID-19 hospitalizations for additionally coded obesity as well as identify independent predictors of in-hospital death of obese COVID-19 patients.

### Study Oversight and Support

There was no commercial support regarding our present study and no foreign influence on the preparation of this report. Since our study did not contain direct access by us (as the investigators) to individual patient data and we had only access to summarized results provided by the RDC, approval by an ethics committee as well as patients’ informed consent was not required, in accordance with German law (26, 27).

### Coding of Diagnoses, Procedures, and Definitions

Shortly after the beginning of the century (since the year 2004), diagnosis- and procedure-related remunerations were introduced and implemented in the German healthcare system for German hospitals. Coding according to the German Diagnosis Related Groups (G-DRG) system with the coding of patient data on diagnoses, coexisting conditions, and surgeries as well as diagnostic and interventional procedures is required, and the transfer of these codes to the Institute for the Hospital Remuneration System is mandatory for German hospitals to get their remuneration regarding rendered and provided services (15, 16). In this context, patients’ diagnoses are coded according to the International Statistical Classification of Diseases and Related Health Problems (of the 10th revision with German modification, ICD-10-GM) (19, 20). In addition, diagnostic, interventional, and surgical procedures are coded according to special OPS codes [Operationen- und Prozedurenschlüssel (surgical and procedural coding). With this present analysis of the German nationwide inpatient sample, we were able to identify all patients with confirmed COVID-19 diagnosis (ICD code U07.1] hospitalized in German hospitals during the year 2020 (COVID-19 as main or secondary diagnosis).

To obtain data of coexisting conditions, comorbidities, complications, and treatments, the aforementioned available diagnostic and procedural codes were used for acute and chronic conditions (OPS and ICD-10-GM codes).

The selected COVID-19 patients were stratified for additionally coded obesity (defined as body mass index [BMI] ≥30 kg/m^2^ according to the WHO; ICD code E66). Patients with obesity were further classified in mild obesity (obesity class I: BMI 30 to <35 kg/m^2^, ICD codes E66.00, E66.10, E66.20, E66.80, and E66.90), moderate obesity (obesity class II: BMI 35 to <40 kg/m^2^, ICD codes E66.01, E66.11, E66.21, E66.81, and E66.91), and severe obesity (obesity class III: BMI >40 kg/m^2^, ICD codes E66.02, E66.12, E66.22, E66.82, and E66.92).

Post-COVID was defined as the status of previous survived COVID-19 infection before the patient’s hospitalization with the actual (and therefore at this time recurrent) COVID-19 infection.

### Study Outcomes and Adverse In-Hospital Events

The primary study outcome was defined as case fatality with death due to all causes during in-hospital stay (in-hospital case fatality). In addition, we analyzed the prevalence of major adverse cardiovascular and cerebrovascular events [MACCE, composite of all-cause in-hospital death, acute myocardial infarction (ICD code I21), and/or ischemic stroke (ICD code I63)] as well as that of the adverse in-hospital events ARDS (ICD code J80), VTE (ICD codes I26, I80-I82), acute renal failure (ICD code N17), myocarditis (ICD code I40), myocardial infarction (ICD codes I21-I22), ischemic or hemorrhagic stroke (ICD codes I61-I64), cardiopulmonary resuscitation (CPR, OPS code 8-77), ICU, OPS codes 8-980, 8-98d, and 8-98f), MV (OPS codes 8-71), gastrointestinal bleeding (ICD code K92.0-K92.2), intracerebral bleeding (ICB; ICD code I61), and transfusion of blood constituents (OPS codes 8-800).

### Statistical Analysis

For the objective, to compare COVID-19 patients with and without obesity, we analyzed the differences between these two groups. The differences in patient characteristics between the groups of hospitalized COVID-19 patients with and without obesity were calculated with the help of the Wilcoxon–Whitney U test for continuous variables and Fisher’s exact or chi (2) test for categorical variables, as appropriate. We used Bonferroni’s correction method for multiple testing. For the reported differences between patient characteristics and adverse in-hospital events of both groups (COVID-19 cases with and without obesity) (presented in **Table 1**), Bonferroni’s correction method for multiple testing was used and indicated that only p-values <0.00139 and not p-value <0.05 identified a significant difference.

**Table 1:** Patients’ characteristics, medical history, presentation, and adverse in-hospital events of the 176,137 hospitalized patients with confirmed COVID-19 infection in Germany in the year 2020 stratified for obesity.

Parameters	COVID-19 with obesity (n = 9,383; 5.3%)	COVID-19 without obesity (n = 166,754; 94.7%)	p-Value*
**Age**	66.0 (54.0/76.0)	72.0 (56.0/82.0)	**<0.001**
**Age ≥ 70 years**	3,879 (41.3%)	90,450 (54.2%)	**<0.001**
**Female sex**	4,704 (50.1%)	79,245 (47.5%)	**<0.001**
**In-hospital stay (days)**	10.0 (5.0/19.0)	8.0 (4.0/14.0)	**<0.001**
**Cardiovascular risk factors**			
**Diabetes mellitus**	4,293 (45.8%)	40,939 (24.6%)	**<0.001**
**Essential arterial hypertension**	5,510 (58.7%)	76,970 (46.2%)	**<0.001**
**Hyperlipidemia**	2,066 (22.0%)	25,507 (15.3%)	**<0.001**
**Comorbidities**			
**Coronary artery disease**	1,692 (18.0%)	23,882 (14.3%)	**<0.001**
**Heart failure**	2,293 (24.4%)	24,826 (14.9%)	**<0.001**
**Peripheral artery disease**	438 (4.7%)	5,202 (3.1%)	**<0.001**
**Atrial fibrillation/flutter**	2,147 (22.9%)	32,013 (19.2%)	**<0.001**
**Chronic obstructive pulmonary disease**	1,077 (11.5%)	11,077 (6.6%)	**<0.001**
**Chronic renal insufficiency (glomerular filtration rate <60 ml/min/1,73 m^2^)**	2,010 (21.4%)	25,362 (15.2%)	**<0.001**
**Cancer**	394 (4.2%)	8,607 (5.2%)	**<0.001**
**Mild liver disease**	276 (2.9%)	1,369 (0.8%)	**<0.001**
**Severe liver disease**	389 (4.1%)	3,750 (2.2%)	**<0.001**
**Charlson comorbidity index**	4.44 ± 3.01	4.08 ± 2.92	**<0.001**
**Respiratory manifestations of COVID-19 and post-COVID-19 status**			
**Pneumonia**	6,505 (69.3%)	100,408 (60.2%)	**<0.001**
**Acute respiratory distress syndrome**	1,464 (15.6%)	10,130 (6.1%)	**<0.001**
**Multi-systemic inflammatory syndrome COVID-19 infection**	46 (0.5%)	451 (0.3%)	**<0.001**
**Post-COVID-19 status**	33 (0.4%)	524 (0.3%)	0.529
**Treatment**			
**Intensive care unit**	2,826 (30.1%)	24,227 (14.5%)	**<0.001**
**Mechanical ventilation**	1,438 (15.3%)	10,704 (6.4%)	**<0.001**
**Extracorporeal membrane oxygenation (ECMO)**	257 (2.7%)	1,197 (0.7%)	**<0.001**
**Dialysis**	648 (6.9%)	4,927 (3.0%)	**<0.001**
**Adverse events during hospitalization**			
**In-hospital death**	1,585 (16.9%)	30,022 (18.0%)	**0.006**
**Major adverse cardiac and cerebrovascular events (MACCE)**	1,788 (19.1%)	33,236 (19.9%)	**0.039**
**Cardiopulmonary resuscitation**	288 (3.1%)	2,571 (1.5%)	**<0.001**
**Venous thromboembolism**	432 (4.6%)	4,555 (2.7%)	**<0.001**
**Acute kidney failure**	2,037 (21.7%)	20,038 (12.0%)	**<0.001**
**Myocarditis**	17 (0.2%)	209 (0.1%)	0.141
**Myocardial infarction**	174 (1.9%)	2,579 (1.5%)	**0.019**
**Stroke (ischemic or hemorrhagic)**	175 (1.9%)	3,021 (1.8%)	0.706
**Intracerebral bleeding**	49 (0.5%)	527 (0.3%)	**0.001**
**Gastrointestinal bleeding**	167 (1.8%)	2,781 (1.7%)	0.410
**Transfusion of blood constituents**	1,129 (12.0%)	12,745 (7.6%)	**<0.001**

^*^After using Bonferroni’s correction method for multiple testing, p-values <0.00139 remain significant.

^*^Significant P-values are marked in bold.

Since COVID-19 is a new disease with the first cases in Germany diagnosed in January 2020 and a learning process regarding pathomechanism, risk factors, and treatments in the years 2020 (1, 2), it is of outstanding interest to analyze the time trends of these patients. Temporal trends of the total numbers of hospitalizations of COVID-19 patients, obesity classes, treatment in ICUs, use of MV, VTE events, and in-hospital mortality over time and with increasing age were estimated by means of linear regression analyses. Results were presented as β-estimates and 95% CIs.

Univariate and multivariate logistic regression models were computed in order to investigate associations between obesity and adverse in-hospital events and invasive treatments. The multivariate regression models were adjusted for age, sex, cancer, heart failure, coronary artery disease, peripheral artery disease, chronic obstructive pulmonary disease, essential arterial hypertension, hyperlipidemia, renal insufficiency (glomerular filtration rate [GFR] <60 ml/min/1,73 m^2^), DM, and atrial fibrillation/flutter. We selected this epidemiological approach regarding this adjustment to test the widespread independence of obesity as an influencing factor on adverse in-hospital events of these outstanding known predictors of case-fatality rate during hospitalization. The results were presented as odds ratios (ORs) and 95% CI. Regarding the logistic regression models, only the p-values <0.05 (two-sided) were considered to be statistically significant.

All statistical analyses were carried out with the use of SPSS software (IBM Corp. Released 2011. IBM SPSS Statistics for Windows, Version 20.0. IBM Corp: Armonk, NY, USA).

## Results

### Baseline Characteristics

Overall, 176,137 hospitalization cases with confirmed COVID-19 infection were reported in Germany during the year 2020. Among them, 9,383 (5.3%) were additionally coded with obesity.

### Comparison of Obese vs. Non-Obese COVID-19 Inpatients

COVID-19 patients without obesity were in median 6 years older (72.0 [interquartile range (IQR) 56.0/82.0] vs. 66.0 [54.0/76.0] years, p < 0.001), while gender distribution was almost equal. Length of in-hospital stay was longer in obese than in non-obese COVID-19 patients (10.0 [5.0/19.0] vs. 8.0 [4.0/14.0], p < 0.001). Despite the younger age of obese COVID-19 patients, all investigated CVD risk factors and CVD were more prevalent in obese COVID-19 patients (**Table 1**). In addition, also chronic obstructive pulmonary disease, liver diseases, and CKD were more frequent in obese COVID-19 patients, and consequently, the Charlson comorbidity index score was higher in obese than in non-obese COVID-19 patients (mean and SD: 4.44 ± 3.01 vs. 4.08 ± 2.92, p < 0.001). In contrast, cancer (5.2% vs. 4.2%, p < 0.001) was more prevalent in non-obese patients (**Table 1**).

**Figure 1:**
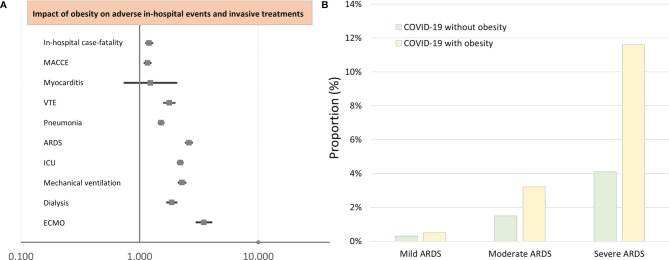
Impact of obesity on adverse in-hospital events and interventional treatments of COVID-19 patients (multivariate logistic regression analysis). **(A)** Impact of obesity on adverse in-hospital events and interventional treatments of COVID-19 patients (multivariate logistic regression analysis). **(B)** Proportion of COVID-19 patients with and without obesity on ARDS. MACCE, major adverse cardiac and cerebrovascular events; VTE, venous thromboembolism; ARDS, acute respiratory distress syndrome; ICU, intensive care unit; ECMO, extracorporeal membrane oxygenation.

The majority of obese and non-obese COVID-19 patients revealed pneumonia as a respiratory manifestation, whereby pneumonia (69.3% vs. 60.2%, p < 0.001) and also ARDS (15.6% vs. 6.1%, p < 0.001) occurred more often in obese than in non-obese COVID-19 patients (**Table 1**). In all ARDS severity categories, obese patients were more frequently detected (**Figure 1B**). Post-COVID-19 status was not more common in one of the groups. After Bonferroni’s correction method was used for multiple testing, all of these reported differences between patient characteristics and adverse in-hospital events of both groups (COVID-19 cases with and without obesity) remained significant, since after Bonferroni’s correction all p-values <0.00139 were still significant.

### Treatment Differences of Obese vs. Non-Obese COVID-19 Inpatients

Obese COVID-19 patients were more often treated on ICUs (30.1% vs. 14.5%, p < 0.001) and needed more often MV (15.3% vs. 6.4%, p < 0.001) (**Table 1**). Extracorporeal membrane oxygenation (ECMO) was more frequently performed in obese patients (2.7% vs. 0.7%, p < 0.001), and the necessity of dialysis was also more than doubled in obese COVID-19 patients (**Table 1**).

### Outcomes of Obese vs. Non-Obese COVID-19 Inpatients and Impact of Obesity on Adverse In-Hospital Events of COVID-19 Inpatients

Despite the unfavorable comorbidity profile of obese COVID-19 patients, the in-hospital case-fatality rate (16.9% vs. 18.0%, p = 0.006), the MACCE rate (19.1% vs. 19.9%, p = 0.039), and rate of myocardial infarction were lower than in non-obese patients, triggered by a large proportion of COVID-19 patients in older age decades of life (**Table 1**). While stroke and myocarditis were similar prevalent in both groups, VTE (4.6% vs. 2.7%, p < 0.001) and acute kidney failure (21.7% vs. 12.0%, p < 0.001) were more frequent in obese patients. Transfusion of blood constituents (12.0% vs. 7.6%, p < 0.001) and ICB events (0.5% vs. 0.3%, p = 0.001) were more often counted in obese patients (**Table 1**).

**Figure 2:**
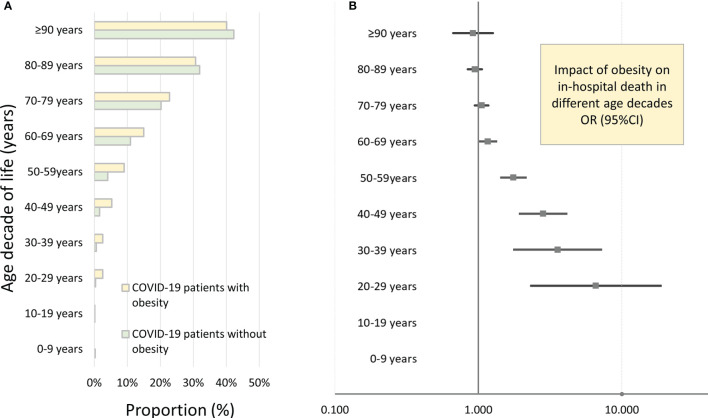
Impact of obesity on in-hospital death of inpatients with COVID-19 infection in Germany 2020 stratified for age decades. **(A)** In-hospital case-fatality rates of COVID-19 patients in different age decades stratified for presence of obesity. **(B)** Independent impact of obesity on in-hospital case fatality of inpatients with COVID-19 infection stratified for age decades (results of the multivariate logistic regression model).

After adjustment for age, gender, and comorbidities, obesity was independently associated with increased in-hospital case fatality (OR 1.203 [95% CI 1.131–1.279], p < 0.001) and MACCE (OR 1.168 [95% CI 1.101–1.239], p < 0.001) rate (**Table 2** and **Figure 1A**), whereas univariate logistic regressions did not reveal the same associations due to clear differences in age, CVD risk factors, and comorbidities between the two groups (COVID-19 patients with and without obesity). For further in-depth analysis, we conducted an age-dependent comparison of COVID-19 patients with and without obesity in each decade of life. The in-hospital case-fatality rate was higher in obese than in non-obese COVID-19 patients in the 3rd to 8th decades of life. In younger patients, case fatality was similar between both groups, and in COVID-19 patients aged 80 years or older, case fatality was not negatively influenced by obesity (**Table 3** and **Figure 2**). The multivariate regression models demonstrated an independent association of obesity with increased case-fatality rate in COVID-19 patients aged between 20 and 69 years. Case-fatality rates of older patients were not significantly and independently influenced. The largest effect of obesity on case-fatality rate was seen in COVID-19 patients in the 3rd life decade with a 6.6-fold increased case-fatality rate (**Table 3** and **Figure 2**).

**Table 2:** Impact of obesity on in-hospital death and adverse events during in-hospital stay in patients with COVID-19 (univariate and multivariate logistic regression models).

	Univariate regression model		Multivariate regression model	
	OR (95% CI)	p-Value	OR (95% CI)	p-Value
In-hospital death	0.926 (0.876–0.978)	**0.006**	1.203 (1.131–1.279)	**<0.001**
MACCE	0.946 (0.897–0.997)	**0.039**	1.168 (1.101–1.239)	**<0.001**
Pneumonia	1.493 (1.428–1.562)	**<0.001**	1.517 (1.448–1.589)	**<0.001**
ARDS	2.858 (2.694–3.033)	**<0.001**	2.605 (2.449–2.772)	**<0.001**
Venous thromboembolism	1.719 (1.554–1.901)	**<0.001**	1.780 (1.605–1.973)	**<0.001**
Acute renal failure	2.030 (1.929–2.137)	**<0.001**	1.955 (1.850–2.066)	**<0.001**
Myocardial infarction	1.203 (1.030–1.404)	**0.020**	0.991 (0.843–1.165)	0.915
Cardiopulmonary resuscitation	2.022 (1.787–2.288)	**<0.001**	1.695 (1.492–1.926)	**<0.001**
Stroke (ischemic or hemorrhagic)	1.030 (0.883–1.201)	0.706	1.013 (0.866–1.185)	0.868
Intracerebral bleeding	1.656 (1.235–2.221)	**0.001**	1.611 (1.194–2.172)	**0.002**
Gastrointestinal bleeding	1.068 (0.913–1.251)	0.410	1.093 (0.931–1.284)	0.277
Transfusion of blood constituents	1.653 (1.549–1.763)	**<0.001**	1.484 (1.387–1.589)	**<0.001**
Intensive care unit treatment	2.536 (2.421–2.655)	**<0.001**	2.201 (2.097–2.310)	**<0.001**
Mechanical ventilation	2.639 (2.486–2.800)	**<0.001**	2.277 (2.140–2.422)	**<0.001**
ECMO	3.895 (3.39–4.464)	**<0.001**	3.485 (3.023–4.017)	**<0.001**
Dialysis	2.437 (2.239–2.652)	**<0.001**	1.869 (1.708–2.045)	**<0.001**
Myocarditis	1.446 (0.882–2.372)	0.144	1.234 (0.746–2.040)	0.413
Post-COVID status	1.120 (0.787–1.593)	0.530	1.079 (0.755–1.543)	0.675

MACCE, major adverse cardiovascular and cerebrovascular events; ARDS, acute respiratory distress syndrome; ECMO, extracorporeal membrane oxygenation.

Significant P-values are marked in bold.

**Table 3:** Impact of obesity on in-hospital death in patients with COVID-19 stratified for age decades (univariate and multivariate logistic regression models).

	Univariate regression model		Multivariate regression model	
	OR (95% CI)	p-Value	OR (95% CI)	p-Value
0–9 years	Not applicable	**-**	Not applicable	**-**
10–19 years	Not applicable	**-**	Not applicable	**-**
20–29 years	7.409 (2.947–18.626)	**<0.001**	6.581 (2.331–18.581)	**<0.001**
30–39 years	4.188 (2.222–7.894)	**<0.001**	3.566 (1.776–7.160)	**<0.001**
40–49 years	3.427 (2.449–4.795)	**<0.001**	2.821 (1.944–4.094)	**<0.001**
50–59 years	2.321 (1.944–2.771)	**<0.001**	1.753 (1.442–2.132)	**<0.001**
60–69 years	1.434 (1.267–1.622)	**0.020**	1.162 (1.017–1.327)	**0.027**
70–79 years	1.166 (1.052–1.294)	**0.004**	1.054 (0.944–1.176)	0.353
80–89 years	0.943 (0.845–1.053)	0.298	0.944 (0.843–1.059)	0.327
90–99 years	0.912 (0.668–1.246)	0.565	0.917 (0.667–1.261)	0.959

Significant P-values are marked in bold.

In addition, obesity affected the occurrence of pneumonia (OR 1.517 [95% CI 1.448–1.589], p < 0.001), ARDS (OR 2.605 [95% CI 2.449–2.772], p < 0.001), and VTE (OR 1.780 [95% CI 1.605–1.973], p < 0.001) (**Table 2** and **Figure 1A**).

Obesity in COVID-19 patients is a risk factor for ICU treatment (OR 2.201 [95% CI 2.097–2.310], p < 0.001), MV (OR 2.277 [95% CI 2.140–2.422], p < 0.001), and the rescue treatment with ECMO (OR 3.485 [95% CI 3.023–4.017], p < 0.001) (**Table 2** and **Figure 1A**).

In addition, a higher rate of acute kidney failure (OR 1.955 [95% CI 1.850–2.066], p < 0.001) resulted in an increased necessity of dialysis treatment (OR 1.869 [95% CI 1.708–2.045], p < 0.001) in patients with obesity. COVID-19 patients presented with an increased risk for ICB (OR 1.611 [95% CI 1.194–2.172], p = 0.002) and had an elevated risk regarding transfusion of blood constituents (OR 1.484 [95% CI 1.387–1.589], p < 0.001) (**Table 2** and **Figure 1A**).

### Impact of Obesity Classes on Outcomes and Treatment

**Figure 3:**
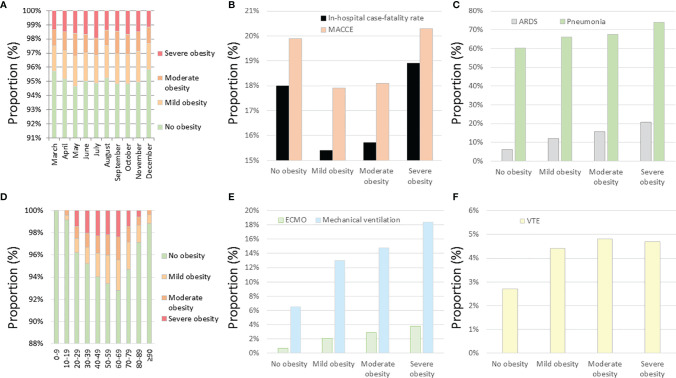
Temporal trends regarding obesity classes and impact of obesity classes on outcomes and treatments of inpatients with COVID-19 infection in Germany 2020. **(A)** Temporal trends regarding obesity classes in inpatients with COVID-19 infection stratified for months. **(B)** Impact of obesity classes on in-hospital case fatality and MACCE in inpatients with COVID-19 infection. **(C)** Impact of obesity classes on ARDS and pneumonia in inpatients with COVID-19 infection. **(D)** Temporal trends regarding obesity classes in inpatients with COVID-19 infection stratified for age decades. **(E)** Impact of obesity classes on ECMO and mechanical ventilation in inpatients with COVID-19 infection. **(F)** Impact of obesity classes on VTE events in inpatients with COVID-19 infection. MACCE, major adverse cardiovascular and cerebrovascular events; ARDS, acute respiratory distress syndrome; ECMO, extracorporeal membrane oxygenation; VTE, venous thromboembolism.

The distribution of the obesity classes of the admitted patients with COVID-19 did not vary significantly over the observational period (**Figure 3A**). The highest proportions of severe obesity were found in the 4th to 7th decades of life (**Figure 3D**). The prevalence of pneumonia, ARDS, and VTE events is increased with increasing obesity classes (**Figures 3C, F**). In contrast, the proportion of COVID-19 inpatients who suffered from MACCE or died during the in-hospital stay was the highest in severe obesity and revealed the lowest mortality in the mild obesity class (**Figure 3B**). Remarkably, the use of MV and ECMO increased with the increase in obesity class (**Figure 3E**).

**Figure 4:**
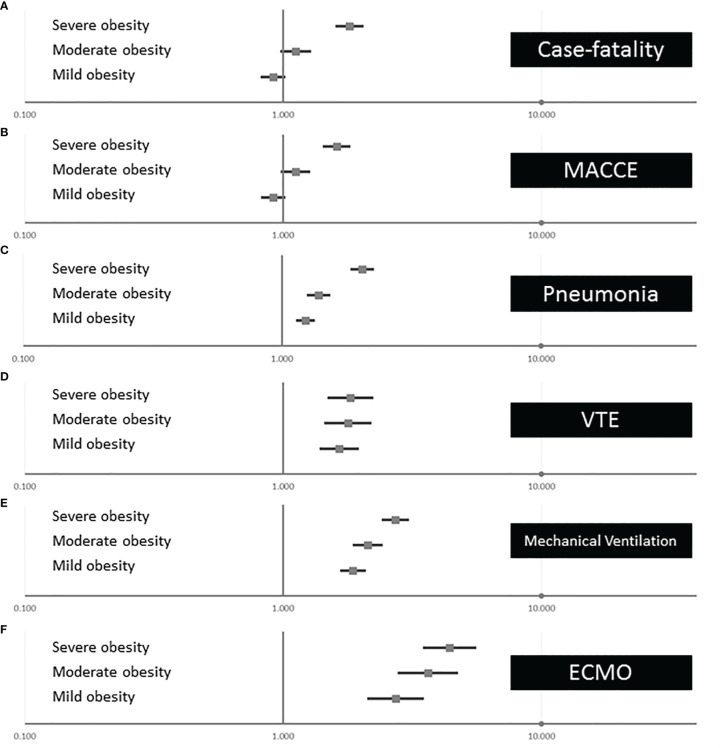
Associations of obesity classes with outcomes and treatments of inpatients with COVID-19 infection in Germany 2020 (multivariate logistic regression models). **(A)** Independent association between the different obesity classes on in-hospital case fatality of inpatients with COVID-19. **(B)** Independent association between the different obesity classes on MACCE of inpatients with COVID-19. **(C)** Independent association between the different obesity classes on pneumonia of inpatients with COVID-19. **(D)** Independent association between the different obesity classes on VTE of inpatients with COVID-19. **(E)** Independent association between the different obesity classes on mechanical ventilation of inpatients with COVID-19. **(F)** Independent association between the different obesity classes on ECMO of inpatients with COVID-19. MACCE, major adverse cardiovascular and cerebrovascular events; VTE, venous thromboembolism; ECMO, extracorporeal membrane oxygenation.

The adjusted multivariate logistic regression models showed that severe obesity was independently associated with case fatality of COVID-19 patients (OR 1.810 [95% CI 1.615–2.029], p < 0.001), whereas mild and moderate obesity affected the case fatality insignificantly (**Figure 4A**). Similarly, MACCE was independently influenced only by severe obesity (OR 1.616 [95% CI 1.447–1.805], p < 0.001), but not from mild and moderate obesity (**Figure 4B**). In contrast, the occurrence of pneumonia and VTE as well as the use of the treatments with MV and ECMO was affected by all obesity classes and the intensity of this impact of obesity on pneumonia, VTE, MV, and ECMO increased with the increase in obesity class (**Figures 4C–F**).

### Temporal and Seasonal Trends

**Figure 5:**
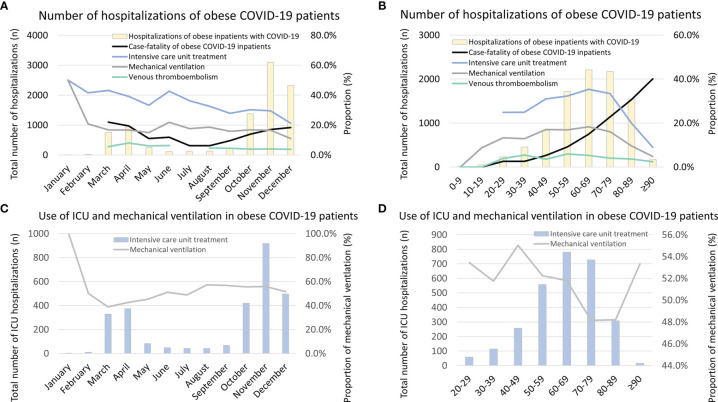
Temporal trends regarding total numbers, case-fatality rate, VTE rate, treatment of ICU, and mechanical ventilation in obese inpatients with COVID-19 infection in Germany 2020. **(A)** Temporal trends regarding total numbers, case-fatality rate, VTE rate, treatment rates of ICU, and mechanical ventilation in obese inpatients with COVID-19 infection stratified for months. **(B)** Temporal trends regarding total numbers, case-fatality rate, VTE rate, treatment rates of ICU, and mechanical ventilation in obese inpatients with COVID-19 infection stratified for age decades. **(C)** Temporal trends regarding total numbers of ICU treatment, and among these, rate of mechanical ventilation in obese inpatients with COVID-19 infection is stratified for months. **(D)** Temporal trends regarding total numbers of ICU treatment, and among these, rate of mechanical ventilation in obese inpatients with COVID-19 infection is stratified for age decades. VTE, venous thromboembolism; ICU, intensive care unit.

The highest monthly numbers of hospitalizations of COVID-19 patients with obesity were observed from October to December 2020 (**Figure 5A**). In November 2020, more than 3,000 obese COVID-19 patients were treated in German hospitals. During summer, the lowest obese COVID-19 patient numbers were detected (**Figure 5A**). The age-dependent analysis showed the highest total numbers of obese COVID-19 patients in the 7th and 8th age decades (**Figure 5B**).

The monthly case-fatality rates were increased in the months with higher numbers of admitted COVID-19 patients during March and April as well as between October and December (**Figure 5B**) but did not change significantly during the observational period when considering a trend over the whole year 2020 (β −0.135 [95% CI −0.304 to 0.034], p = 0.116). In contrast, the in-hospital case-fatality rate of obese COVID-19 patients increased exponentially with age (β 1.041 [95% CI 0.957 to 1.125], p < 0.001) **(** **Figure 5B**). Remarkably, the necessity of ICU treatment (β −0.909 [95% CI −1.045 to −0.882], p < 0.001) and MV (β −0.323 [95% CI −0.499 to −0.147], p < 0.001) decreased over the months of the year 2020 (**Figure 5A**).

The proportion of obese COVID-19 patients who were treated in ICUs and had to be ventilated was highest in the 5th to 8th life age decades (**Figure 5B**). Statistically, patient numbers of ICU treatment (β −0.120 [95% CI −0.191 to −0.050], p = 0.001) and MV (β −0.159 [95% CI −0.249 to −0.069], p = 0.001) decreased with increasing age decades (**Figure 5B**). In **Figure 5C**, the total numbers of obese COVID-19 patients treated in ICUs stratified for the different months are shown. The figure illustrates that the monthly proportions of ventilated patients who were admitted to ICUs were the highest in January and February 2020 and decreased afterward, although the total numbers of obese COVID-19 patients in ICUs were small in the first 2 months of 2020 compared to later months. This might be caused and may represent a learning curve regarding the ICU treatment modalities in German hospitals (**Figure 5C**). Although the total numbers of COVID-19 patients with obesity admitted to German ICU between 20th and 49th life-years were low in comparison to the age group between 50th and 79th life-years, the proportion of MV was substantially higher in the younger age group (**Figure 5D**).

Last but not least, the monthly numbers of VTE events decreased over time (β −0.823 [95% CI −1.125 to −0.522], p < 0.001) (**Figure 5A**) but remained widely unchanged with age (β −0.128 [95% CI −0.283 to 0.027], p = 0.105) (**Figure 5B**).

## Discussion

One wave after another of the COVID-19 pandemic has been affecting the citizens and healthcare systems of the countries worldwide. At the end of January 2022, nearly 350 million COVID-19 cases and more than 5.5 million deaths linked with a COVID-19 infection were counted worldwide (10). Regarding risk stratification and better management of these patients with COVID-19 infections, it is of outstanding interest to identify risk factors of adverse outcomes and in-hospital death in hospitalized patients with COVID-19 to initiate adequate treatment, prevent poor outcomes, and save hospital resources to manage pandemic waves without overwhelming regional healthcare systems (2, 5). Several studies identified, among others, obesity is one of these risk factors for higher proneness to SARS-CoV-2 infection and a poorer outcome during COVID-19 infection (2, 10, 12, 14–17).

Thus, in the present study, we attempted to analyze the impact of obesity on adverse in-hospital events in all hospitalized COVID-19 patients in Germany during the year 2020.

The main results of the study can be summarized as follows:

i) COVID-19 patients with obesity were distinctly younger at admission; nevertheless, obese COVID-19 patients presented with an aggravated comorbidity profile.
ii) The majority of obese and non-obese COVID-19 patients presented with pneumonia as their respiratory manifestation, whereby pneumonia and ARDS occurred more frequently in obese than in non-obese COVID-19 patients.
iii) Obese COVID-19 patients were treated more often admitted to ICUs with a higher rate of organ support, such as MV, dialysis, and ECMO.
iv) Obesity was independently associated with increased in-hospital case fatality and MACCE rate as well as with pneumonia and VTE events. Particularly, severe obesity was associated with case fatality as well as MACCE.
v) An age-dependent impact of obesity on in-hospital case fatality was observed with the highest impact in the 3rd and 4th decades of life, whereas obesity in older patients (≥8th decade of life) did not affect in-hospital case fatality independently.

In accordance with the literature (18, 28), our study results demonstrated that COVID-19 inpatients with obesity were younger than those without. Despite younger age, obese patients were affected with an unfavorable comorbidity profile, including CVD risk factors and CVD, and also lung diseases and CKD, similarly to other studies (13, 15, 16, 22, 28–31).

Our study results highlighted that the majority of COVID-19 patients suffered from pneumonia as their respiratory manifestation of COVID-19, whereby pneumonia and ARDS were more prevalent in obese than in non-obese COVID-19 patients. Regardless of the pathophysiological effects of acute respiratory failure (ARF) and ARDS, obese patients are characterized by multiple alterations in respiratory mechanics. The compliance of the respiratory system is influenced by the external compression due to the weight of fat tissue resulting in an impaired functional residual capacity (FRC) (32–34). The obesity hypoventilation syndrome is a well-known problem in severe obesity also outside COVID-19 infection and ICU treatment and is associated with an increase regarding the need for ventilator support and organ damage (35, 36).

Regarding the setting of respiratory parameters, the identification of optimal positive end-expiratory pressure (PEEP) in COVID-19 pneumonia and associated ARDS has especially been widely discussed since the beginning of the pandemic (37). Current consensus recommendations are provided on the basis of the ARDS network table to detect adequate PEEP (38). Since these recommendations for PEEP settings were derived from experiences in the standard population of patients predominantly with normal weight, it has to be suggested that obese patients might be ventilated with significantly higher PEEP values in order to overcome external compression of the respiratory system. Therefore, alternative approaches are needed to identify the optimal individualized PEEP in obese COVID-19 patients, such as electric impedance tomography or transpulmonary pressure measurement (39). In this context, too low PEEP values can result in negative end-expiratory trans-pulmonary pressure levels accompanied with elevated risk regarding the occurrence of atelectasis, impaired gas exchange, and cyclic lung collapse followed by pulmonary inflammation and ARF (40, 41).

Some other cornerstones in the up-to-date treatment of ARDS in COVID-19 patients, such as prone positioning, are also harder to achieve in obese patients. Prone positioning in obese patients requires more staff (nurses, respiratory therapists (RTs), and physicians) to provide it safely. Nevertheless, prone positioning could in severe obesity be harmful, and its implementation in the individual ICU treatment could sometimes be impossible if not provided by a skilled team (42–44).

In addition to the changes in respiratory mechanics, recent studies have indicated that the occurrence, disease severity of COVID-19 patients, and the attributed adverse outcomes of COVID-19 patients are directly associated with the weakened immune system in obese individuals by chronic inflammation due to adipose tissue and by dysregulation of pro-inflammatory cytokines (13, 15, 45). Therefore, it has to be hypothesized that acute inflammation arising from acute COVID-19 infection may amplify existing chronic inflammation secondary to obesity and might lead to a more severe disease status as well as poorer outcomes (15).

Our study results corroborate the aforementioned physiological effects of obesity, demonstrating that obese COVID-19 patients in Germany revealed more severe disease status and were treated more often in ICUs and with a higher rate of MV, dialysis, and ECMO. This more aggressive treatment approach might be attributed to lower patient age and the higher number of obese COVID-19 patients who were treated at larger urban hospitals with more invasive capacities. However, it has been previously reported that obese patients have a higher demand for more aggressive treatments in the context of acute and critical care medicine including especially invasive treatment approaches (46–50).

Despite these strong efforts in therapy, obesity in COVID-19 was independently associated with increased in-hospital case-fatality rate and MACCE rate as well as VTE events in our study in accordance with others (10, 12, 14–17). Although the unadjusted comparison between obese and non-obese COVID-19 patients revealed that obese COVID-19 patients had a 1.1% lower in-hospital case-fatality rate (16.9% vs. 18.0%) in comparison to non-obese COVID-19 patients, the adjusted logistic regressions revealed a negative effect of obesity on the case-fatality rate. The multivariate logistic regression models (regarding the outcome in-hospital case fatality) demonstrated eminently an association of obesity in COVID-19 patients with increased in-hospital case fatality independently of age, sex, cancer, heart failure, coronary artery disease, peripheral artery disease, chronic obstructive pulmonary disease, essential arterial hypertension, hyperlipidemia, renal insufficiency (GFR < 60 ml/min/1,73 m^2^), DM, and atrial fibrillation/flutter. For a more precise and in-depth comparison, we performed logistic regression models to analyze the impact of obesity on the case-fatality rate of COVID-19 patients in each decade of life. The age-dependent analysis demonstrated an independent association of obesity with increased case-fatality rate in the 3rd, 4th, 5th, 6th, and 7th decades of life in COVID-19 patients, while obesity did not affect case-fatality rates of older patients significantly and independently. Remarkably, the effect of obesity on the case-fatality rate was larger in COVID-19 patients in the 3rd and 4th decades of life. Since most hospitalized COVID-19 patients were older, it is not surprising that the univariate logistic regressions failed in the overall study sample without age-adjustment or age-dependent analysis to demonstrate an increased in-hospital case fatality in the unadjusted comparisons and was only evident in the adjusted and age-dependent analyses.

Gao et al. highlighted in accordance with our results that even below the threshold for obesity, a BMI higher than 23 kg/m^2^ was associated with an increased risk of severe COVID-19 outcomes, particularly in patients younger than 40 years (13, 16). Also in line with these observations, the study of Mohammad et al. identified obesity as a major risk factor for adverse outcomes in patients with COVID-19 (15). In a large Swedish cohort of ICU patients with COVID-19, a high BMI was associated with an increased risk of death and prolonged length of in-hospital stay as well (18). Tartof et al. reported in a retrospective study that obesity plays an important role in risk for death from COVID-19, especially in male and younger patients (17). In addition, a large Korean study revealed a non-linear (U-shaped) relationship between BMI and fatal illness, meaning that especially patients with underweight as well as having a BMI ≥25 kg/m^2^ had a higher risk of fatal COVID-19 disease (28). Also an analysis of the Premier Healthcare data of the United States (March–December 2020) showed that obesity was a risk factor for MV, hospitalization, and death predominantly among adults aged <65 years (51).

Based on nationally representative data on demographics and the cardiometabolic conditions of the patients, O’Hearn et al. estimated that nearly 2/3 (63.5%) of COVID-19 hospitalizations among the adult citizens of the United States were attributable to four cardiometabolic conditions: obesity, DM, arterial hypertension, and heart failure. Within this group, obesity (30.2%) and arterial hypertension (26.2%) were linked to most COVID-19 hospitalizations (52). The large epidemiology study of Popkin et al. reported an increase of COVID-19 patients’ hospitalizations, ICU admissions, and deaths in nearly 400,000 COVID-19 patients with obesity worldwide (53). In contrast to some of the studies, our study results demonstrated that particularly severe obesity affected in-hospital case fatality and MACCE rate independently and significantly, while mild obesity and moderate obesity were not independently associated with these outcomes. In addition, as aforementioned, the impact of obesity was age-dependent.

In one previous publication of our research group, we observed a temporal trend regarding hospitalizations and in-hospital case-fatality rate of all hospitalized patients with COVID-19 infection in Germany 2020 (2). In line with the time trend of all admitted COVID-19 patients in Germany, the highest monthly numbers of hospitalizations of COVID-19 patients with obesity were observed during October to December 2020 and in the 7th and 8th age decades of life. Remarkably, the case-fatality rate was higher in the months with increased numbers of admitted COVID-19 patients, which might be interpreted as a surrogate of overloading of the healthcare system, and increased exponentially with age (2, 5, 8, 9).

Several pathophysiological mechanisms of obesity as a risk factor driving severe COVID-19 illness have been suggested (19): first, the adipose tissue has more angiotensin-converting enzyme-2 receptors, which is the site for the coronavirus entry into the cells, than does the lung tissue, so the excess adipose tissue could serve as a reservoir for the coronavirus (29, 30). Also an excess of abdominal fat and weight impairs adequate ventilation and increases the risk for infection (including expansion of the COVID-19 infection but also secondary infections) (19, 31), which might explain higher rates of pneumonia and ARDS of obese patients in our study. Certainly, central or abdominal obesity or visceral adipose tissue may carry a particularly “heavy” risk in COVID-19. Ventilating obese patients *via* mask ventilation or intubation is more difficult not least because of an increased thoracic mass accompanied by a need for higher positive end-expiratory and peak pressures to maintain proper oxygenation and an increased risk of barotrauma (such as alveolar injury/rupture and pneumothorax) (19, 31). Since our study detected a decrease regarding the necessity of ICU admissions and MV over the months of the year 2020, this could be interpreted as a learning curve of the treating physicians regarding the use of invasive treatments in obese COVID-19 patients. As mentioned, the effect of a weakened immune system in obese individuals by chronic inflammation due to adipose tissue and by dysregulation of pro-inflammatory cytokines might predispose obese COVID-19 patients to more severe infections (13, 15, 19, 45). Last but not least, social factors may contribute to poor outcomes, including loneliness, and might be followed by a higher frequency of depressive syndromes (19). In addition, because the focus is on the healthcare system and the physicians working with the COVID-19 pandemic, other prevalent acute or chronic diseases that carry an increased risk for adverse outcomes in patients with COVID-19 infection and severe COVID-19 complications might be overlooked, be considered to be unimportant at this time, or be unnoticed (19). National lockdown measures with stay-at-home orders for longer periods favored sedentary lifestyle with distinctly reduced physical activity (PA) and exacerbated unhealthy dietary habits resulting in increased prevalence of obesity during the pandemic along with the psychological effects of social isolation (5, 8, 9, 19, 54). Nevertheless, the proportion of COVID-19 cases with severe obesity did not change over the months (**Figure 3A**), indicating only a slow shift regarding more severe obesity.

Obesity predisposes patients to develop a VTE event, which was reported by Karbhel et al., who demonstrated a strong association between increasing BMI and higher VTE rate (55). The authors outlined that the relative risk of unprovoked pulmonary embolism (PE) increased by approximately 8% per 1 kg/m^2^ higher BMI and approached an approximately 6-fold elevated risk among individuals with a BMI ≥ 35 kg/m^2^ (55, 56). This finding was supported by the results of the present study, revealing a 1.7-fold risk for VTE in obese vs. non-obese patients. VTE is a potentially life-threatening complication, and hospitalized COVID-19 patients frequently have macrovascular and microvascular thrombosis and inflammation, which are associated with a poor clinical outcome (57, 58). Interestingly, the number of VTE events in obese COVID-19 patients decreased over time but remained unchanged with age. This is an important finding, since the risk of VTE events increased in the general population, substantially with age (27, 59–61). Besides the challenges in dosing anticoagulants in critical ill overweight patients, it should be suggested that some VTE events might be overlooked since not all COVID-19 patients were examined by CT with angiography and PE might be fatal in COVID-19 patients before PE is diagnosed (62).

Based on our findings, we recommend that COVID-19 patients with obesity should be closely monitored when hospitalized (18). The awareness regarding VTE events is important in obese patients; these events or delays in the diagnosis of these life-threatening complications should not be overlooked (27, 59, 62). When clinicians develop healthcare plans for COVID-19 patients, they should consider the risk of poor outcomes, especially in patients with severe obesity (51). In addition, these considerations should be included in the promotion of COVID-19 prevention strategies and healthcare planning (51, 63, 64). Maintaining a healthy weight is therefore important not only to prevent chronic cardiometabolic diseases but also regarding better individual outcomes of COVID-19 patients (28, 63, 65, 66). Thus, public health efforts directed towards a healthy diet and an increase in PA are crucial in both children and adults (19, 63, 64, 66–70). A body of evidence suggests that the nutritional status of COVID-19 patients is directly associated with the severity of the SARS-CoV-2 disease (71–73). Although no single diet or food item has been proven to prevent COVID-19 infections, some key dietary components comprising vitamins C and D, omega-3 fatty acids, and zinc might have positive anti-inflammatory effects similar to the Mediterranean diet and might enhance the health of COVID-19 patients (71–73). In addition, the protective role of nutraceuticals such as quercetin and resveratrol in patients with obesity or cancer has to be considered and emphasized for regularization of the hypersecretion of interleukins and cytokines in order to improve the immune function and reduce the risk of ARDS and inflammation (73, 74). International efforts are needed to prevent obesity, particularly its progression to more severe degrees, and to improve PA, exercise, and fitness, as well as overall healthy living, for this and future pandemics (29, 31, 63–70).

### Limitations

It is well known that obesity is an increasing problem worldwide (75, 76). The proportion of adults with prevalent obesity was estimated at 21.9% in male and 22.5% in female citizens of Germany (75). Thus, a rate of 5.3% obese COVID-19 patients suggests that there may have been an under-reporting and under-coding of obesity in the nationwide sample and, particularly, in those patients who died during the first hours after admission. Nevertheless, the rates in the nationwide sample of the United States and Korea were similar to ours (28, 77), despite a prevalence of obesity of 42% in adults in recent analyses of the United States (19, 78).

## Conclusions

Obesity independently affected case fatality, MACCE, VTE, and other adverse in-hospital events of COVID-19 patients. Obesity should be taken into account regarding COVID-19 prevention strategies, risk stratification, and adequate healthcare planning. Maintaining a healthy weight is important not only to prevent chronic cardiometabolic diseases but also for better individual outcomes during COVID-19 infection.

## Data Availability Statement

The original contributions presented in the study are included in the article/supplementary material, further inquiries can be directed to the corresponding author/s.

## Ethics Statement

Ethical review and approval were not required for the study on human participants in accordance with the local legislation and institutional requirements. Written informed consent from the participants’ legal guardian/next of kin was not required to participate in this study in accordance with the national legislation and the institutional requirements.

## Author Contributions

Conceptualization: KK and LH. Writing—original draft: KK. Writing—review and editing: KK, IS, VHS, VS, CE-K, CL, TM, and LH. Data curation: KK and LH. All authors discussed the results and contributed to the final manuscript.

## Conflict of Interest

TM is PI of the DZHK (German Center for Cardiovascular Research), Partner Site Rhine-Main, Mainz, Germany. CE-K reports having received fees from Amarin Germany, Amgen GmbH, Bayer Vital, Boehringer Ingelheim, Bristol-Myers Squibb, Daiichi Sankyo, Leo Pharma, MSD Sharp & Dohme, Novartis Pharma, Pfizer Pharma GmbH, and Sanofi-Aventis GmbH. LH received lecture/consultant fees from MSD and Actelion, outside the submitted work.

The remaining authors declare that the research was conducted in the absence of any commercial or financial relationships that could be construed as a potential conflict of interest.

## Publisher’s Note

All claims expressed in this article are solely those of the authors and do not necessarily represent those of their affiliated organizations, or those of the publisher, the editors and the reviewers. Any product that may be evaluated in this article, or claim that may be made by its manufacturer, is not guaranteed or endorsed by the publisher.
